# Indigenous Probiotic *Lactobacillus* Isolates Presenting Antibiotic like Activity against Human Pathogenic Bacteria

**DOI:** 10.3390/biomedicines5020031

**Published:** 2017-06-16

**Authors:** Debashis Halder, Manisha Mandal, Shiv Sekhar Chatterjee, Nishith Kumar Pal, Shyamapada Mandal

**Affiliations:** 1 Laboratory of Microbiology and Experimental Medicine, Department of Zoology, University of Gour Banga, Malda 732103, India; debashishalder1991@gmail.com; 2 Department of Physiology, MGM Medical College and LSK Hospital, Kishanganj, Bihar 855107, India; debmanisha@rediffmail.com; 3 Department of Microbiology, NRS Medical College and Hospital, Kolkata 700014, India; shivshibu@rediffmail.com (S.S.C.); pal_nishith@yahoo.co.in (N.K.P.)

**Keywords:** lactobacilli, probiotics, antagonistic activity, indicator bacterial pathogens, antibiotic susceptibility, γ-hemolytic activity, cumulative probiotic potential

## Abstract

Background: Indigenous lactic acid bacteria are well known probiotics having antibacterial activity against potentially pathogenic bacteria. This study aims to characterize the curd lactobacilli for their probiotic potentiality and antagonistic activity against clinical bacteria. Methods: Four curd samples were processed microbiologically for the isolation of lactic acid bacteria (LAB). The LAB strains obtained were identified by conventional methods: cultural aspect, gram-staining, biochemical and sugar fermentation tests. The probiotic properties were justified with tolerance to low-pH, bile salt and sodium chloride, and the antagonistic activity of the lactobacilli against human pathogenic bacteria (*Escherichia coli*, *Proteus vulgaris*, *Acinetobacter baumannii* and *Salmonella enterica* serovar Typhi) was assessed. Hemolytic activity and antibiotic susceptibility were determined for the lactobacilli isolates, and the cumulative probiotic potential (CPP) values were recorded. Result: Four lactobacilli isolates, *L. animalis* LMEM6, *L. plantarum* LMEM7, *L. acidophilus* LMEM8 and *L. rhamnosus* LMEM9, procured from the curd samples, survived in low-pH and high bile salt conditions, and showed growth inhibitory activity against the indicator bacteria by agar-well (zone diameter of inhibition; ZDIs: 13.67 ± 0.58–29.50 ± 2.10 mm) and agar overlay (ZDIs: 11.33 ± 0.58–35.67 ± 2.52 mm) methods; the average growth inhibitory activity of lactobacilli ranged 233.34 ± 45.54–280.56 ± 83.67 AU/mL, against the test bacterial pathogens. All the lactobacilli were non-hemolytic and sensitive to most of the test antibiotics. The CPP values of the isolated LAB were recorded as 80–100%. Conclusion: The curd lactobacilli procured might be used as the valid candidates of probiotics, and bio-therapeutics against bacterial infection to humans.

## 1. Introduction

Among the lactic acid bacteria (LAB), lactobacilli (the species of the genus *Lactobacillus*) are the principal members of the intestinal microbiota of vertebrates, including humans, and involve themselves in fermentation of various foods, thereby improving the food quality and safety, and the health and comfort of the consumers as well. Such microorganisms are generally recognized as safe (GRAS) and can be used safely as probiotics [1]. Their adaptation to two different environments, the extra-intestinal environment such as food, and the human intestine (in terms of colonization and persistence), exerting varied selective pressure related to their growth rate and carbohydrate metabolism (viz., the food isolates of *L. reuteri* capable of maintaining host-specific physiological characteristics) considered them as probiotics [2,3,4]. The bacterial (pathogenic) multidrug resistance and formation of biofilm lead to the lack of effectiveness of antibiotics available in the treatment of infection, while the administration of probiotics has been seen functional in preventing and/or counteracting the biofilm-related infection [5]. However, the antibacterial activity of probiotic lactobacilli appears to be strain-specific [6]. Because they act against pathogenic bacteria in the gastrointestinal tract or in the food through multifunctional ways, by secreting antimicrobial substances (H_2_O_2_, lactic acid and other organic acids and bacteriocins) [7], competing for nutrients and binding sites, or counteracting the spread within the colonized body [8], probiotic bacteria have the capacity to maintain and influence the composition of the healthy intestinal microbiota [7].

Products containing lactobacilli dominate the global probiotics market; from a wide range of sources, they are used widely in biotechnology and food preservation, and are being explored as therapeutics [9]. It has been demonstrated that the *Lactobacillus plantarum* isolates could protect the infection caused with *Salmonella typhi* through interference with growth as well as virulence properties (adherence, invasion, and cytotoxicity) of the pathogen [7]. A large number of lactobacilli isolates, procured from traditional fermented foods prepared with the combination of cereals and dairy materials, including *L. acidophilus*, *L. plantarum* and *L. rhamnosus* had excellent antibacterial activity against *Escherichia coli* ATCC 700728 standard strain [10]. The lactic acid bacterial strains *L. acidophilus*, *L. plantarum*, *L. fermentum*, *L. casei* and *L. rhamnosus*, can effectively be applied against the urinary tract infection causing *Proteus* species (*P. mirabilis* and *P. vulgaris*), as has been reported by Goudarzi et al. [11]. In the earlier study, utilizing some curd lactobacilli and commercially available lactobacilli strains, we demonstrated, singly and in combination, their antibacterial activity against *Klebsiella pneumoniae*, and *E. coli* [12].

The current study was undertaken to evaluate the probiotic potentiality and antagonistic effect of four natural lactobacilli strains, procured from various locally available commercial curds and homemade cow milk curds, on gram-negative pathogenic bacteria (*Salmonella enterica* serovar Typhi, *Proteus vulgaris*, *K. pneumoniae*, and *E. coli*) causing infection to humans.

## 2. Materials and Methods

### 2.1. Curd Samples and Lactic Acid Bacteria

A total of four curd samples, commercial curd 1 (open sample prepared from cow milk), home-made cow milk curd, commercial curd 2 (sachet sample prepared from cow milk) and commercial curd 3 (cup sample prepared from cow milk), were utilized in the current study. In order to isolate the lactic acid bacteria (LAB), de Man, Rogosa and Sharpe (MRS) broth (Hi-Media, Mumbai, India) was inoculated with freshly collected curd samples, and after incubation for 24–48 h at 35 °C, single discrete colonies were procured on MRS agar (Hi-Media, India) plate, from each of the curd samples, by streak dilution of the broth culture as described earlier [13]. The each of the bacterial colonies isolated was identified performing conventional culture and gram-staining, and physiological and biochemical (catalase, oxidase, indole, nitrate, TSI, MR-VP) tests including sugar fermentation, following Bergey’s manual [14], as described earlier [13].

### 2.2. Probiotic Property

The probiotic properties of the isolated lactobacilli were determined through tolerance to sodium chloride, bile salt and low-pH. The bile salt and low-pH (acid) tolerance was tested following the protocol of Liong and Shah [15], and to sodium chloride, by using the protocol of Chowdhury et al. [16], with slight modifications as mentioned elsewhere [13]. Briefly, the isolated lactobacilli were grown (for 24 h at 37 °C), in MRS broth with sodium chloride supplementation of 2, 4 % and 6.5 %, and thereafter the growth of lactobacilli, following subculture of the MRS broth cultures, on MRS agar (for 24 h at 37 °C), indicated their tolerance to sodium chloride. The tests were conducted under atmospheric carbon dioxide, and were replicated twice.

### 2.3. Antagonistic Activity

The antagonistic activity of four lactobacilli isolates procured (designated as LMEM6, LMEM7, LMEM8 and LMEM9) was determined by agar overlay and agar-well diffusion method, against the indicator strains: *Salmonella enterica* serovar Typhi (*S. typhi* from blood culture), *Escherichia coli* (*E. coli* from urine culture), *Proteus vulgaris* (*P. vulgaris* from pus culture) and *Acinetobacter baumannii* (*A. baumannii* from pus culture), procured from different clinical samples, as has been mentioned herein.

#### 2.3.1. Agar Overlay Method

The *Lactobacillus* isolates were spot inoculated, separately, onto the MRS agar plates, using a loop-full (≈10^5^ CFU/spot) of MRS broth culture (grown at 35 °C for 48 h) of the lactobacilli, and the inoculated plates were incubated at 37 °C for 48 h. The MRS agar plates containing the growth of lactobacilli in spot form (5 mm diameter) were thereafter overlaid with soft Muller-Hinton agar (0.8% agar) pre-mixed with 10^8^ CFU of the indicator stains (one on each MRS agar plate), and incubated, after solidification of the overlaid agar medium, at 37 °C for 24 h. The zone diameter of inhibition (ZDI) values obtained were measured and interpreted following Shokryazdan et al. [17]: the ZDI >20 mm, 10–20 mm and <10 mm were considered as strong, intermediate and weak inhibitions, respectively. The “*R*” (width of clear zone) values were also determined as per the formula stated earlier [12]: $R=\frac{\left(d\;\mathrm{Inhib}\;–\;d\;\mathrm{Spot}\right)}{2}$ (“*d* Inhib”: the diameter of clear zone around the “*d* Spot”; and “*d* Spot”: the diameter of spot form of lactobacilli grown on MRS agar plate). The scores of growth inhibition of indicator bacteria were considered as no inhibition capacity when “*R*” was <2 mm; low inhibition capacity with “*R*” values of 2–5 mm, and high inhibition capacity with “*R*” values >6 mm [18,19]. All tests were repeated thrice and the data were represented as mean ± SD (standard deviation).

#### 2.3.2. Agar-Well Diffusion Method

The agar-well method was performed according to Tagg and McGiven [20]. On the surface of nutrient agar plate swabbed with indicator bacterial broth culture, wells (of 6 mm diameter) were prepared, and culture filtrates (75 µL/well) of the isolated lactobacilli were loaded in the wells marked properly with the isolates’ names. Following 24 h incubation at 37 °C (in presence of atmospheric CO_2_), ZDI (zone diameter of inhibition) values (nearest whole) were recorded, and interpreted as less active, moderately active and highly active with ZDIs ≤10 mm, 11–14 mm, and ≥15 mm, respectively. The tests were performed thrice and the data were represented with mean ± SD. The antagonistic activity of the test lactobacilli in arbitrary unit per mL(AU/mL) was calculated (mean ± SD) as a measure of production of bioactive components using the formula mentioned elsewhere [21]: $\mathrm{AU/mL}=\frac{ZDI\;\times \;1000}{\mathrm{Volume}\;\mathrm{taken}\;\mathrm{in}\;\mathrm{the}\;\mathrm{well}\;\left(\mu \text{L}\right)}$, where ZDI denotes “zone diameter of inhibition”.

### 2.4. Safety Profiling

The safety profile of the curd isolates of *Lactobacillus* (LMEM6, LMEM7, LMEM8 and LMEM9) was determined by their hemolytic activity and antibiotic susceptibility.

#### 2.4.1. Hemolytic Activity

For hemolytic activity, the overnight grown MRS broth culture of the lactobacilli strains were streaked on blood agar plate (Hi-Media, India) and incubated at 37 °C for 72 h; thereafter, the plates were observed for the formation of any clean (β-hemolysis) or greenish (α-hemolysis) hemolytic zones, or no such zone (γ-hemolysis) around the *Lactobacillus* colonies.

#### 2.4.2. Antibiotic Susceptibility

The antibiotic susceptibility test was performed following disc diffusion method [22], as described before by Halder and Mandal [12], using MRS agar (Hi-Media, Mumbai, India), and approximately 10^8^ CFU inocula from the four lactobacilli strains [23]. The antibiotic discs (Hi-Media, Mumbai, India) used were amoxyclav (Ax: 30-µg/disc), ampicillin (Am: 10-µg/disc), gentamicin (Gm: 30-µg/disc), tetracycline (Tc: 30-µg/disc) and vancomycin (Vm: 30-µg/disc). The ZDI values (nearest whole in three consecutive repeats) obtained were interpreted according to the criteria mentioned earlier [24,25]: the lactobacilli were grouped into resistant (ZDI: ≤15 mm), sensitive (ZDI: ≥21 mm), or intermediately susceptible (ZDI: 16–20 mm).

### 2.5. Cumulative Probiotic Potential

The probiotic potential of the *Lactobacillus* isolates was assessed using 5 point scores, and the cumulative probiotic potential (CPP) was calculated as per the formula: $CPP=\frac{Observed\;score}{Maximum\;score}\times 100$, depicted by Tambekar and Bhutada [26].

## 3. Results

Four lactic acid bacteria, one from each of the four curd samples, were isolated: LMEM6 (from commercial curd 1), LMEM7 (from home-made cow milk curd), LMEM8 (from commercial curd 2) and LMEM9 (from commercial curd 3). All isolates were gram-positive non-spore forming non-motile rod shaped and were negative to catalase and oxidase tests, and thus recognized as *Lactobacillus*. Following morphological, cultural, biochemical tests and sugar fermentation profile, the isolates were identified as *L. animalis* LMEM6, *L. plantarum* LMEM7, *L. acidophilus* LMEM8 and *L. rhamnosus* LMEM9. The tolerance test results to different stresses (sodium chloride, low-pH and bile salts) for the isolated lactobacilli are represented in Table 1.

**Figure 1:**
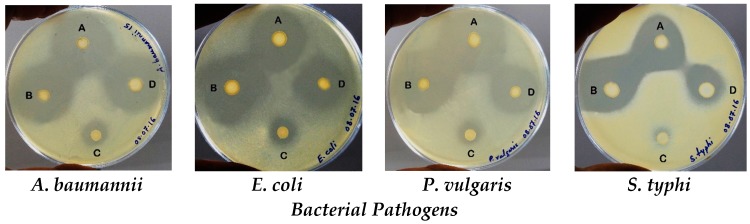
Agar overlay technique demonstrates the antibacterial activity of natural lactobacilli isolates against four bacterial pathogens used as the indicator strains. The zones of inhibition have been seen around four test lactobacilli strains (grown on the plates as spot forms) against each of the four bacterial pathogens (*A. baumanni*, *E. coli*, *P. vulgaris* and *S. typhi*): (**A**) *L. animalis* LMEM6; (**B**) *L. plantarum* LMEM7; (**C**) *L. acidophilus* LMEM8; and (**D**) *L. rhamnosus* LMEM9.

The curd lactobacilli isolates showed excellent antibacterial activity, following agar overlay method, against all indicator bacteria tested (Figure 1). The *L. plantarum* LMEM7 isolate had top growth inhibitory activity against *A. baumannii*, *E. coli* and *P. vulgaris* having ZDIs 32.33 ± 0.58, 30.00 ± 1.71 and 35.67 ± 2.52 mm, respectively, while the *S. typhi* showed highest sensitivity to *L. animalis* LMEM6 isolate (ZDI: 30.50 ± 0.71 mm); the *L. acidophilus* LMEM8 isolate had poor activity against all indicator bacterial strains with ZDIs 11.33 ± 0.58 to 15.25 ± 0.96 mm (Table 2).

The “*R*” values of the isolated lactobacilli against the gram-negative pathogenic bacteria have been represented in Table 3. The lowest “*R*” values (3.17 ± 0.29–5.13 ± 0.48 mm) were recorded due to the action of *L. acidophilus* LMEM8 isolate, while the values, ranging from 10.13 ± 0.85 to 15.33 ± 1.26 mm, were from the action of *L. plantarum* LMEM7 isolate against the pathogenic bacteria tested; the *L. rhamnosus* LMEM9 had “*R*” values 7.83 ± 0.76–13.17 ± 0.76 mm, and *L. animalis* LMEM6 had 14.75 ± 0.35 mm against *P. vulgaris*.

The antagonistic activity, following agar-well diffusion (based on the ZDIs values obtained around the culture filtrate-loaded wells on the agar plates), of the lactobacilli isolates against the indicator bacteria is represented in Table 4. The *L. animalis* LMEM6 had highest activity, with ZDI of 23.67 ± 1.53 mm, against *P. vulgaris*, while *A. baumannii*, *E. coli* and *S. typhi* were highly sensitive to *L. rhamnosus* LMEM9, having ZDIs 20.33 ± 1.53, 29.50 ± 2.10 and 20.00 ± 1.00 mm, respectively. Among the curd lactobacilli, the *L. acidophilus* LMEM8 and *L. rhamnosus* LMEM9 had lowest activity (182.22 ± 7.70–191.11 ± 20.37 AU/mL), among the pathogenic indicator bacterial isolates, while the top level of growth inhibitory components produced was 393.34 ± 27.76 AU/mL, by *L. rhamnosus* LMEM9, and the values ranged from 195.56 ± 7.70 to 315.55 ± 20.37 AU/mL, for *L. animalis* LMEM6, and from 204.45 ± 20.37 to 263.34 ± 25.24 AU/mL, for *L. plantarum* LMEM7 (Table 5).

The isolated lactobacilli (*L. animalis* LMEM6, *L. plantarum* LMEM7 and *L. acidophilus* LMEM8 and *L. rhamnosus* LMEM9) had no clear transparent or greenish zone on the blood agar plates, surrounding their colonies, and thus were found γ-hemolytic or non-hemolytic. The antibiotic susceptibility test results for the curd lactobacilli are shown in Table 6. All the *Lactobacillus* isolates had resistance to Vm, and *L. animalis* LMEM6 had Ax resistance, in addition to the Vm. The all isolated curd lactobacilli showed sensitivity to Tc, while sensitivity to Am and Ax was shown by *L. plantarum* LMEM7 and *L. acidophilus* LMEM8; the *L. animalis* LMEM6 was also sensitive to Am and Gm. The intermediately susceptibility (IS) to Gm was recorded for *L. plantarum* LMEM7, *L. acidophilus* LMEM8 and *L. rhamnosus* LMEM9 isolates, with IS for *L. rhamnosus* LMEM9 to Am too.

The individual CPP for the *Lactobacillus* isolates was 80% for *L. animalis* LMEM6, and 100% for the rest three isolates: *L. plantarum* LMEM7, *L. acidophilus* LMEM8 and *L. rhamnosus* LMEM9 (Table 7).

## 4. Discussion

Probiotics, which are otherwise called the beneficial gut bacteria, have become a “popular therapy” in recent years, and the researchers are investigating how probiotic microorganisms, viz., *Lactobacillus* interact with the body [27]. In addition, the isolation and screening of lactobacilli from various locally available natural sources is a victorious way to develop new improved probiotic strains with precious medical relevance, though plentiful commercial probiotic strains are currently available in markets worldwide. In the present study four lactobacilli from different curd samples have been procured and their probiotic capacity has scientifically been validated. Samuel et al. [28] isolated a large number of lactic acid bacteria from various food samples (dosa batter, raw milk, curd and paneer), and confirmed their species level identity, based on the cultural, morphological and biochemical characteristics, as *L. casei*, *L. plantarum*, *L. fermentum*, *L. brevis*, *L. acidophilus*, *L. bulgaricus* and *L. rhamnosus.* Following the criteria mentioned above, the isolated curd lactobacilli in our study have been designated as *L. animalis* LMEM6, *L. plantarum* LMEM7, *L. acidophilus* LMEM8 and *L. rhamnosus* LMEM9.

It has been demonstrated that for probiotic characterization of lactobacilli, acid tolerance is an important criterion, and the pH value of 3.0 has been considered standard for such investigation of probiotic strains [29,30]. As per the report of Shokryazdan et al. [17], the entire test *Lactobacillus* isolates had tolerance to acid at pH 3.0, for 3 h. The *L. plantarum* strains isolated from fermented olives have been reported to survive for a period of 2–6 h at pH 2.0 and pH 3.0 [31]. Ehrmann et al. [32] documented that various lactobacilli, including *L. animalis*, tolerated acidic condition, at pH 3.0, for 4 h; however, the tolerance level varied among the isolates. The *Lactobacillus* strains retained the viability when exposed them to acidic environment at the pH values of 2–3 [7]. In earlier studies, as have been conducted by Jose et al. [33] and Liu et al. [34], the lactobacilli tolerated and survived in MRS broth (pH: 3), while reduction in viability has been seen at pH 2. The *Lactobacillus* strains isolated from river buffalo milk cheese showed survivability in presence of NaCl (1–7%), indicating their high sodium chloride tolerance [35]. The buffalo milk probiotic isolates of *L. fermentum* and *L. acidophilus* survived luxuriously in presence of 1–6% NaCl, whereas moderate growth was seen at 7% concentration of NaCl [36]. Regarding bile salt tolerance of probiotic lactobacilli, their survivability in presence of 0.3% bile salt is physiologically significant, since the bile salts at such a level achieve normally in the human intestine [37], or, differently, the 0.3% bile salts mimic the physiological bile concentration [38]. As per the investigation of Koll et al. [30], the test strains of *Lactobacillus* had tolerance to 0.3% bile. Rahman [36] reported the growth and survivability of *L. fermentum* and *L. acidophilus* isolates from buffalo milk at the bile salt concentrations of 0.3–0.5%. It has been reported, similar to our observation, elsewhere that the lactobacilli were seen to grow luxuriously as well as to survive well in 0.3% of bile salt supplement, whereas poor tolerance was recorded at 0.2% bile salt condition [33,39]. The varied levels of tolerance to NaCl, bile salts and low pH (pH: 2–4), have been demonstrated in the four lactobacilli of the current study: *L. animalis* LMEM6, *L. plantarum* LMEM7, *L. acidophilus* LMEM8 and *L. rhamnosus* LMEM9.

The conception of antagonism of pathogenic bacteria using probiotic lactobacilli has been well documented, and the antibacterial property of such friendly microorganisms has been considered as an important attribute in selecting potential probiotics for the maintenance of healthy microbial balance in the gut. The LAB, mostly the lactobacilli, possessing the capacity to alienate the bacterial pathogens through the production of some antimicrobials, such as H_2_O_2_, organic acids (mainly, lactic acid), bacteriocin, acquires the enviable property for probiotic potentiality and a maintainable substitute to the synthetic antibiotics. The *Lactobacillus* isolates (*L. plantarum*, *L. fermentum* and *L. salivarius*) from fermented fruits and vegetables had broad antibacterial spectrum (ZDI: 26–28 mm), against food-borne bacterial pathogens, as has been reported by Manzoor et al. [40]. A large number of lactobacilli isolates, including *L. acidophilus*, *L. plantarum* and *L. rhamnosus*, procured from traditional fermented foods prepared with the combination of cereal and dairy materials, had excellent antibacterial activity against *E. coli* with ZDIs 17–20, 21–30, 14–31 mm, respectively [10].The probiotic *Lactobacillus* strains: *L. fermentum*, *L. casei* and *L. acidophilus*, isolated from buffalo milk showed growth inhibitory activity against *Vibrio cholerae*, *S. typhi*, *E. coli*, and *Shigella* species having ZDIs 10–22 mm [36]. The lactobacilli strains, including *L. rhamnosus* and *L. plantarum*, procured from dairy food products (commercially available yoghurt and cheese) and rumen contents of cow did not show growth inhibitory activity against *E. coli*, while *Salmonella menston* was found sensitive to all the test lactobacilli, as per the demonstration of Jose et al. [33]. The supernatant of lactic acid bacteria from the ecological niches of Ecuador, following agar well diffusion, at low pH conditions (pH: 3.0–4.0) had ZDI > 15 mm, against the target bacterial pathogens [41]. Thus, the antagonism of pathogenic bacteria with the lactobacilli is strain/isolate as well as pathogen (target bacteria) specific. The antibacterial antagonism test results, when compared with the two methods (agar overlay and agar-well diffusion) employed in the current study, was in accordance with the results reported by Cadirci and Citak [42], who inspected antagonism of LAB against gram-negative bacteria using the above two methods and found the spot method (agar overlay method) as the effective one in the evaluation of inhibitory activity [42]. However, Rahimifard and Naseri [43] demonstrated well diffusion method as the best to determine antagonism than the other two methods (disk diffusion and spot on lawn) employed, utilizing the probiotic strain, *Bifidobacterium bifidum* against *Salmonella enterica* serovar Enteritidis. The lactobacilli from curd samples herein had excellent antibacterial activity in agar-well (ZDI: 13.67 ± 0.58–29.50 ± 2.10 mm) as well as agar overlay (ZDI: 11.33 ± 0.58–35.67 ± 2.52 mm) methods, with “*R*” values 3.17 ± 0.29–15.33 ± 1.26 mm. The variation in antibacterial activities as depicted by different authors might be due to the number of CFU of the LAB used (in spot method) and/or the amount of culture supernatant used (in agar well diffusion) as well as the bacteriocin activity (AU/mL) possessed in it. As has been reported by Iyapparaj et al. [21], the bacteriocin production, in terms of antagonistic activity, for the test lactobacilli ranged 410.4–649.2 AU/mL. The *Lactococcus lactic* subspecies *lactis* RM39 showed strong bacteriocin activity (1600 AU/mL) against *Klebsiella pneumoniae* ATCC 12296, while the other four LAB isolates had inhibitory activity of 800 AU/mL against *E. coli* [44]. In the current assay, the average growth inhibitory activity of lactobacilli was recorded as 233.34 ± 45.54–280.56 ± 83.67 AU/mL, against the test bacterial pathogens.

The probiotic microorganisms must be safe, i.e., the probiotic bacteria like lactobacilli, essentially be incompetent to cause hemolysis as well as gelatin liquefaction in host body. In addition, the probiotic LAB be sensitive to antibiotics so as to be inept disseminating the resistance property to other pathogenic bacteria in the same niche, or the antibiotic resistance among them should be innate and non-transferable [33]. The hemolysis and gelatin hydrolysis remain the two major virulence factors among pathogenic bacteria, and hence, as the safety aspects that have been evaluated for the curd lactobacilli in the current study, the in vitro test for hemolytic activity has been considered first; the next one in the probiotic safety issues being the antibiotic susceptibility (gelatin hydrolytic activity for the test lactobacilli was not represented herein though all the isolates were negative for the test). In the instant investigation, not a single one of the four *Lactobacillus* isolates from curd caused α- or β-hemolysis, although α-hemolysis among lactobacilli from foods and dairy products is not uncommon [45]. Georgieva et al. [46] reported the sensitivity of natural isolates of lactobacilli (*L. acidophilus*, *L. brevis*, *L. fermentum* and *L. plantarum*) to Am, Gm, erythromycin (Em) and Tc, and intrinsic resistance to Vm (non-transferable), and suggested their use as probiotics appropriate in clinical practice. Salminen et al. [47] reported too, the Vm resistance as an intrinsic property of lactobacilli, while the *L. plantarum* isolates had been reported to be sensitive to most of the antibiotics tested, such as penicillin G, Am, chloramphenicol (Ch) and ciprofloxacin, and Vm [31]. The test lactobacilli including *L. fermentum* and *L. acidophilus* were sensitive to Am, Gm, kanamycin (Km), streptomycin (Sm), erythromycin (Em), clindamycin (Cm), Tc and Ch [17]. The antibiotic sensitivity as well as the intrinsic antibiotic resistance property of lactobacilli helps formulate safe probiotic products for human consumption [46]. Many of the *Lactobacillus* strains are naturally resistant to Vm raising concerns regarding the transferability of such resistance to other pathogenic bacteria, but, being chromosomally encoded the Vm resistance is not readily transferable from lactobacilli, as the fact has been demonstrated through conjugation experiments [48]. The *L. kefiri* strains though had sensitivity to Tc, clindamycin, Sm, Am, Em, Km, and Gm, the strains CIDCA 8321 and 8345 were resistant to Ch [18]. The current isolates of curd lactobacilli were sensitive to most of the antibiotics tested with a common resistance to Vm.

The demonstration of cumulative probiotic potential (CPP) of the native lactobacilli strains has been considered as an improved criterion for the probiotic validation [26,49]. The CPP of *L. acidophilus* F14, isolated from buttermilk has been recorded as 100% [50]. Tambekar and Bhutada [26] isolated *L. rhamnosus* G119b and *L. plantarum* G95 strains from fermented milk and evaluated probiotic potential with varied CPP values: the value among the isolated *Lactobacilli* was highest (100%) compared to the probiotic preparations (75–85%) available in the market, and the standard strains of LAB (77–81%). The CPP for *L. brevis* UN was reported as 95.83% [49], while 91.7–100% among the lactobacilli isolates from fermented foods found in Himachal Pradesh, India [51]. Gautam and Sharma [49] reported *L. spicheri* G2 strain, isolated from gundruk, as a potential probiotic fulfilling different probiotic criteria with CPP value 95.83%. In the current study, the curd lactobacilli strains had CPP values 80–100%, and with an average of 95%, and thus the native LAB strains meet the criteria of FAO/WHO [52], in determining the status of a safe probiotic.

## 5. Conclusions

The curd lactobacilli procured might be used as the valid candidates of probiotics, and bio-therapeutics against bacterial infection to humans. To the best of our understanding, this is the first study unfolding the antagonistic activity of four lactobacilli, procured from unexplored native biota of curd, employing two different in vitro methods, against gram-negative pathogenic bacteria, and the probiotic potential of such lactic acid bacteria has been authenticated. However, further studies are required to explore the effectiveness of the antibacterial essence of such native lactobacilli, to be used as the alternative therapeutics, in combating bacterial antibiotic resistance and treating the infection.

## Figures and Tables

**Table 1:** Physiological stress tolerance test results (24 h incubation) for curd lactobacilli.

*Lactobacilli* Strains	NaCl (%)			pH			Bile Salt (%)		
	2	4	6.5	2	3	4	0.125	0.25	0.5
*L. animalis* LMEM6	+	+	+	+	+	+	+	+	+
*L. plantarum* LMEM7	+	+	w	−	w	+	+	+	−
*L. acidophilus* LMEM8	+	+	w	−	w	+	+	+	W
*L. rhamnosus* LMEM9	+	+	+	w	w	+	+	+	+

“+”: resistant/tolerant; “−”: sensitive/non-tolerant; “w”: weakly tolerant.

**Table 2:** Antibacterial activity of the isolated lactobacilli, in terms of ZDI, following agar overlay method.

*Lactobacillus* Isolates	ZDI (mm) ± SD for Indicator Bacteria			
	*A. baumannii*	*E. coli*	*P. vulgaris*	*S. typhi*
*L. animalis* LMEM6	29.67 ± 0.58	28.25 ± 0.82	34.50 ± 0.71	30.50 ± 0.71
*L. plantarum* LMEM7	32.33 ± 0.58	30.00 ± 1.71	35.67 ± 2.52	25.25 ± 1.71
*L. acidophilus* LMEM8	15.25 ± 0.96	12.50 ± 1.29	12.50 ± 0.71	11.33 ± 0.58
*L. rhamnosus* LMEM9	31.33 ± 1.53	22.67 ± 1.53	26.00 ± 2.16	20.67 ± 1.53

ZDI: zone diameter of inhibition; SD: standard deviation.

**Table 3:** The “*R*” values of four natural lactobacilli isolates against the indicator bacterial pathogens: *A. baumanni*, *E. coli*, *P. vulgaris* and *S. typhi*.

*Lactobacillus* Isolates	*R* Value (mm), Mean ± SD, for the Pathogenic Indicator Bacteria			
	*A. baumannii*	*E. coli*	*P. vulgaris*	*S. typhi*
*L. animalis* LMEM6	12.3 ± 0.29	11.50 ± 0.41	14.75 ± 0.35	12.75 ± 0.35
*L. plantarum* LMEM7	13.67 ± 0.29	12.63 ± 0.85	15.33 ± 1.26	10.13 ± 0.85
*L. acidophilus* LMEM8	5.13 ± 0.48	3.75 ± 0.65	3.75 ± 0.35	3.17 ± 0.29
*L. rhamnosus* LMEM9	13.17 ± 0.76	8.83 ± 0.76	10.50 ± 1.08	7.83 ± 0.76

“*R*”: zone of clearance; SD: standard deviation.

**Table 4:** Antibacterial activity of natural lactobacilli isolates against the indicator bacterial pathogens following agar-well diffusion method.

*Lactobacillus* Isolates	ZDI (mm) ± SD for Indicator Bacteria			
	*A. baumannii*	*E. coli*	*P. vulgaris*	*S. typhi*
*L. animalis* LMEM6	14.67 ± 0.58	21.67 ± 1.53	23.67 ± 1.53	18.33 ± 1.53
*L. plantarum* LMEM7	16.00 ± 2.16	19.75 ± 1.89	14.67 ± 1.53	19.50 ± 1.30
*L. acidophilus* LMEM8	13.67 ± 0.58	21.00 ± 1.00	15.67 ± 1.53	19.67 ± 0.58
*L. rhamnosus* LMEM9	20.33 ± 1.53	29.50 ± 2.10	14.33 ± 1.53	20.00 ± 1.00

ZDI: zone diameter of inhibition; SD: standard deviation.

**Table 5:** Growth inhibitory activity of lactobacilli expressed in “AU/mL” for bacterial pathogens.

*Lactobacillus* Isolates	Antagonistic Activity (AU/mL)				Average (AU/mL)
	ST	PV	EC	AB	
*L. animalis* LMEM6	244.45 ± 20.37	315.55 ± 20.37	288.89 ± 20.37	195.56 ± 7.70	261.11 ± 52.63
*L. plantarum* LMEM7	260.00 ± 17.21	204.45 ± 20.37	263.34 ± 25.24	213.33 ± 28.81	235.28 ± 30.72
*L. acidophilus* LMEM8	262.22 ± 7.70	208.90 ± 20.37	280.00 ± 13.33	182.22 ± 7.70	233.34 ± 45.54
*L. rhamnosus* LMEM9	266.67 ± 13.34	191.11 ± 20.37	393.34 ± 27.76	271.11 ± 20.37	280.56 ± 83.67

AB: *Acinetobacter baumannii*; EC: *Escherichia coli*; PV: *Proteus vulgaris*; ST: *Salmonella enterica* serovar Typhi. AU/mL = arbitrary unit per mL.

**Table 6:** Antibiotic susceptibility test results for *Lactobacillus* isolates from curd samples.

Strain	R (ZDI: ≤15 mm)	IS (ZDI: 16–20 mm)	S (ZDI: ≥21 mm)
*L. animalis* LMEM6	Vm: 6; Ax: 15	–	Am: 22; Tc 19; Gm: 19
*L. plantarum* LMEM7	Vm: 6	Gm: 18	Ax: 37; Am: 43; Tc: 29
*L. acidophilus* LMEM8	Vm: 6	Gm: 20	Ax: 27; Am: 31; Tc: 31
*L. rhamnosus* LMEM9	Vm: 6	Am: 20; Gm: 18	Ax: 23; Tc: 21

Ax: amoxyclav Am: ampicillin; Gm: gentamicin; Tc: tetracycline; Vm: vancomycin; IS: intermediately susceptible; R: resistant; S: sensitive; ZDI: zone diameter of inhibition.

**Table 7:** Cumulative probiotic potential (CPP) score card for the isolated lactobacilli.

Probiotic Characters	Indicator Score	Individual Probiotic Isolate Score			
		LMEM6	LMEM7	LMEM8	LMEM9
Acidic pH tolerance	Resistant = 1 Sensitive = 0	1	1	1	1
Bile salt tolerance	Resistant = 1 Sensitive = 0	1	1	1	1
Antagonistic activity(Average)	AU/mL 150–< 200 = 0.5; AU/mL ≥ 200 = 1	1	1	1	1
ntibiotic sensitivity	Intrinsic resistance/Sensitive = 1 Other resistance = 0	0	1	1	1
Hemolytic activity	β-hemolytic = 0 α-hemolytic = 0 γ-hemolytic = 1	1	1	1	1
Total score	5	4	5	5	5
CPP for the lactobacilli		80%	100%	100%	100%
